# Roles of Beta2- and Beta3-Adrenoceptor Polymorphisms in Hypertension and Metabolic Syndrome

**DOI:** 10.4061/2010/832821

**Published:** 2010-10-21

**Authors:** Kazuko Masuo

**Affiliations:** Nucleus Network Ltd. and Human Neurotransmitter Laboratory, Baker IDI Heart and Diabetes Research Institute, 89 Commercial Road, Melbourne, VIC 3004, Australia

## Abstract

Hypertension, diabetes mellitus (especially type 2 diabetes mellitus), metabolic syndrome and obesity are rapidly growing public health problems. Sympathetic nerve activation is observed in obesity, hypertension and diabetes mellitus, which have strong genetic as well as environmental determinants. Reduced energy expenditure and resting metabolic rate are predictive of weight gain, and the sympathetic nervous system participates in regulating energy balance through thermogenesis. The thermogenic effects of catecholamines in obesity have been mainly mediated via the *β*2- and *β*3-adrenergic receptors in humans. Further, *β*2-adrenoceptors importantly influence vascular reactivity and may regulate blood pressure. Genetic polymorphistns of the *β*-adrenoceptor gene have been shown to alter the function of several adrenoceptor subtypes and thus to modify the response to catecholamine. *β*2-adrenoceptor polymorphisms (Arg16Gly, Gln27Glu, and Thr164Ile) have been studied in relation to hypertension. Genetic variations in the *β*3-adrenoceptor (i.e. Try64Arg variant) are also associated with both obesity and hypertension. However, the precise relationships of the polymorphisms of *β*2- and *β*3-adrenoceptor genes with sympathetic nervous system activity, hypertension, and metabolic syndrome have not been fully clarified. This paper will discuss the current topics involving the influence of the sympathetic nervous system and *β*2- and *β*3- adrenoceptor polymorphisms in hypertension and metabolic syndrome.

## 1. Introduction

Obesity, hypertension, and metabolic syndrome (type 2 diabetes mellitus) are major and growing health problems and are known as high-risk factors for subsequent cardiovascular and renal complications [1–3]. Obesity, hypertension, diabetes, and metabolic syndrome are intimately associated [4–6], and sympathetic nervous activation is frequently observed in those conditions. Thus, sympathetic nerve activation may play a major role in the onset and development of hypertension, obesity, and metabolic syndrome (diabetes mellitus) as well as cardiovascular complications in patients with hypertension, diabetes and obesity [2, 7].

The sympathetic nervous system plays an important role in the regulation of energy expenditure. Reduced energy expenditure and resting metabolic rate are predictive of weight gain (obesity). The sympathetic nervous system participates in regulating energy balance through thermogenesis [8]. A large part of the sympathetic nervous system-mediated energy expenditure takes place in skeletal muscle, via the coupling of catecholamines with *β*2-adrenoceptors. Catecholamines are also powerful regulators of lipolysis and act via *β*1-, *β*2-, *β*3- (stimulatory), and *α*2- (inhibitory) adrenoceptor subtypes in adipose tissue, where their role becomes especially important during both exercise and energy restriction, when increased need for fat as a fuel exists. Thus, *β*-adrenoceptors play important roles in energy expenditure and control body weight [9–13]. 

Recently, there is evidence that human hypertension and obesity have strong genetic backgrounds [14–16]. Harrap et al. reported that about 46% of the phenotype of systolic blood pressure are determined genetically for hypertension [17, 18]. Masuo et al. [18–22] have reported close relationships between *β*2- and *β*3-adrenoceptor polymorphisms accompanying elevated sympathetic nervous activity, blood pressure elevation (hypertension), weight gain (obesity), and insulin resistance in a series of longitudinal study. Many epidemiological studies on the relationships between *β*-adrenoceptor polymorphisms, hypertension, obesity, and diabetes (metabolic syndrome) have still been discordant. 

This paper will discuss the current topics involving the contribution of the sympathetic nervous system and *β*2- and *β*3-adrenoceptor polymorphisms in the onset and the development of hypertension and metabolic syndrome (type 2 diabetes mellitus).

## 2. Subtypes of Adrenoceptors (Table 1)

The adrenoceptors (or adrenergic receptors) are a class of G protein-coupled receptors which specifically bind their endogenous ligands, the catecholamines (epinephrine and norepinephrine). Many tissues possess these adrenoceptors, and the binding of an agonist generally elicits a “typical” sympathetic response (i.e., the fight-or-flight response). Table 1 shows the effects of catecholamines bound to adrenoceptors (Table 1) and these effects on sympathetic nervous activity are through *α*- and *β*-adrenergic receptors. 

There are several types of adrenergic receptors, but there are two main groups: *α*-adrenoceptors (*α*1- and *α*2-adrenoceptors) and *β*-adrenoceptors (*β*1-, *β*2-, and *β*3-adrenoceptors). Table 1 also summaries the distributions and functions of the *α*1-, *α*2-, *β*1-, *β*2-, and *β*3-adrenoceptors [24, 25]. The *α*-receptors bind norepinephrine and epinephrine, though norepinephrine has higher affinity. Phenylephrine is a selective agonist of the *α*-adrenoceptors (both *α*1- and *α*2-receptors), thus phenylephrine is usually used to investigate the *α*-adrenoceptors function. *β*-adrenoceptors are linked to G proteins, which are linked to adenyl cyclase. *β*-adrenoceptor agonists cause the intracellular elevation of the second messenger cyclic AMP. Downstream effects of cyclic AMP include cyclic AMP dependent protein kinase, which mediates the intracellular events following hormone binding.

## 3. Sympathetic Nervous Activity and Insulin Resistance in Hypertension (Figure 1)

Insulin resistance in hypertension has been well documented in many epidemiological and clinical studies [8, 26, 27]. Several investigators have reported that chronic insulin administration elevates blood pressure in rats and in humans [28], although insulin also has effects on vasodilation. In addition, many clinical and epidemiological studies have demonstrated the close relationships between sympathetic nerve activity, insulin resistance and hypertension [19, 29–32]. 

Landsberg and other investigators examined the effect of feeding and starvation on sympathetic nerve activity in the cardiac tissue of animals, noting that feeding raised sympathetic nerve activity, and starvation had the opposite effect [33–35]. Energy intake stimulates hyperinsulinemia and sympathetic nerve activity resulting in blood pressure elevations in a cycle to inhibit thermogenesis. Insulin-mediated sympathetic nerve stimulation in obese subjects is a compensatory mechanism aimed at restoring the energy balance by increasing the metabolic rate [33]. Therefore, hyperinsulinemia and insulin resistance in obese subjects are all part of a response to limit further weight gain via stimulating sympathetic nerve activity and thermogenesis [28]. 

On the other hand, Julius et al. [36] have hypothesized that increased sympathetic nerve activity in skeletal muscle causes neurogenic vasoconstriction, thereby reducing blood flow to muscle and consequently inducing a state of insulin resistance by lowering glucose delivery and uptake in hypertension and obesity. Both blood pressure elevation and weight gain may reflect a primary increase in sympathetic nervous tone. Masuo et al. [30, 37] supported Julius's hypothesis. They described that high plasma norepinephrine might predict future blood pressure elevations and weight gain accompanying deterioration in insulin resistance observed in HOMA-IR (homeostasis model assessments of insulin resistance) [30, 37]. Rocchini et al. [38] reported that clonidine prevented insulin resistance in obese dogs over a 6-week period. Their results suggest that sympathetic nerve activity might play a major role in the development of insulin resistance accompanying blood pressure elevations. Valentini et al. [39] reported attenuation of hemodynamic and energy expenditure responses to isoproterenol infusion in hypertensive patients, suggesting that sympathetic nerve activity-induced hypertension may subsequently lead to the development of obesity. 

**Figure 1:**
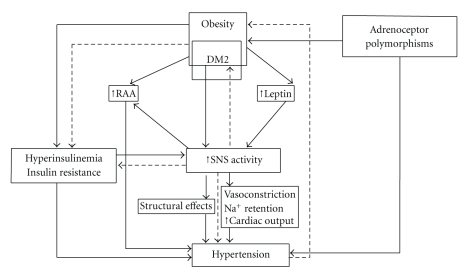
Potential pathophysiological mechanisms by which obesity may contribute to hypertension (modified figure from [23]). RAAS: renin-angiotensin-aldosterone system; SNS: sympathetic nervous system; OSA: obstructive sleep apnea; BRS, baroreflex sensitivity.

Many epidemiological studies showed close linkages of beta2- and beta3-adrenoceptor polymorphisms with obesity, hypertension, and the metabolic syndrome shown in Tables 2, 3, and 4. Sympathetic nervous activity is related to body weight or blood pressure through *β*-adrenoceptors. Thus, close linkages between sympathetic nerve activity and insulin resistance might depend on the *β*-adrenoceptor polymorphisms. Thus, one could speculate that the strong associations between *β*-adrenoceptor polymorphisms and insulin resistance might provide evidence that heightened sympathetic nerve activity followed by insulin resistance might play a major role in hypertension and obesity, because *β*-adrenoceptor polymorphisms might relate to insulin resistance through heightened sympathetic nerve activity (Figure 1).

## 4. Role of *β*-Adrenoceptor Polymorphisms in Hypertension, Obesity, and Diabetes

The sympathetic nervous system plays an important role in the regulation of energy expenditure and blood pressure regulation. A large part of the sympathetic nervous system-mediated energy expenditure takes place in skeletal muscle, via the coupling of catecholamines with *β*2-adrenoceptors. Catecholamines are also powerful regulators of lipolysis and act via *β*1-, *β*2-, *β*3- (stimulatory), and *α*2- (inhibitory) adrenoceptor subtypes in adipose tissue, where their role becomes especially important during both exercise and energy restriction, when increased need for fat as a fuel exists. Stimulation of *β*-adrenergic receptors by the sympathetic nervous system is a significant physiological modulator of pre- and postprandial energy expenditure [11–13] and total daily energy expenditure [9, 10]. 

Recent studies show that *β*-adrenoceptors are polymorphic. Single nucleotide polymorphisms might have functional consequences in terms of receptor activity and regulation and hence may contribute to the pathophysiology of hypertension and obesity. On the other hand, there are few studies on the relationships between *α*-adrenoceptor polymorphisms, hypertension, obesity, and metabolic syndrome. 

### 4.1. *β*1-Adrenoceptor Polymorphisms

The *β*1-adrenoceptor is predominantly expressed in cardiac myocytes and adipose tissue, where its activation leads to increased heart rate and contractility and stimulation of lipolysis, respectively. The two most common *β*1-adrenoceptor polymorphisms are Ser49Gly and Arg389Gly, with relative allele frequencies of 0.85/0.15 and 0.70/0.30 in the Caucasian population, respectively. The *β*1-adrenoceptor is a candidate gene for obesity because of its role in catecholamine-mediated energy homeostasis [72, 73]. For example, in obese individuals, the degree of weight loss during a very low calorie diet has been shown to correlate with changes in *β*1-adrenoceptor protein concentration in adipose tissue [72]. A population cohort of 761 women showed that women carrying the Gly49 genotype had greater increases in BMI over15 years compared to those with the Ser49 genotype [73]. Conversely, the distribution of the Arg389Gly polymorphism is similar in lean and obese subjects [74] and in a large cohort study including 3981 normotensive and 2518 hypertensive subjects [75]. The factors which might explain the discrepancy of published data are shown in the later section.

### 4.2. *β*2-Adrenoceptor Polymorphisms

The *β*2-adrenoceptor is the dominant lipolytic receptor in white human adipose tissue [13] and in skeletal muscle [12]. It also plays an important regulatory role in the peripheral vasculature. Genetic polymorphisms of the *β*2-adrenoceptor have been associated with hypertension, obesity, and metabolic syndrome (diabetes mellitus). The most common polymorphisms are Arg16Gly, with an allele frequency of 0.40/0.60, and Gln27Glu, with an allele frequency of 0.55/0.45, in the Caucasian population. The Thr164Ile polymorphism is rare, occurring in only 3 to 5% of the general Caucasians population. 

Studies of agonist stimulation in cultured cells demonstrate that Gly16 receptors have a greater reduction in numbers or enhanced downregulation when compared with Arg16 whereas the Glu27 receptor is resistant to down regulation when compared with the Gln27 variant [108]. A number of clinical studies have investigated the impact of these polymorphisms on vascular responsiveness [40, 109]. Gratze et al. [110] found that young normotensive white men homozygous for the Gly16 allele had higher blood pressure and lower peripheral vasodilation after infusion of the *β*2-agonist salbutamol. Similar results were obtained by Hoit et al. [111] using the agonist terbutaline. On the other hand, three studies investigating isoprenaline induced increase in the limb blood flow Thus, volunteers homozygous for Gly16 exhibited larger vasodilatory responses than did volunteers homozygous for Arg16 [23]. Conflicting results have also been published with regard to the effects of genetic variants on the sympathetic nervous system modulation of energy expenditure. Bell et al. [112] reported that the response of resting energy expenditure to nonspecific *β*-adrenoceptor stimulation (with isoproterenol infusion) was not different between the 3 genotypes of Arg16Gly. Stob et al. [41] showed that individuals carrying the Arg16Arg variant of the *β*2-adrenoceptor gene have a reduced thermogenic response to selective *β*2-adrenoceptor activation. 

Associations of *β*2-adrenoceptor polymorphisms with hypertension and metabolic syndrome have been reported in many epidemiological studies but results are also discordant (summarised in Tables 2 and 3).

### 4.3. *β*3-Adrenoceptor Polymorphisms

The *β*3-adrenoceptor, which is mainly expressed in adipose tissue, differs from the *β*2-adrenoceptor in two ways: it has a lower affinity for catecholamines, and it resists desensitisation (i.e., downregulation). These characteristic differences might lead to the different effects of catecholamine on *β*2-adrenoceptors and *β*3-adrenoceptors. *β*3-adrenoceptors stimulate the mobilization of lipids from the white fat cell and increase thermogenesis in brown fat cell. Decreased function of *β*3-adrenoceptor in white adipose tissue could slow lipolysis and thereby cause the retention of lipids in fat cells. Slow lipolysis may contribute strongly to visceral obesity in human, and treatment of obese animal models with selective *β*3-adrenergic agonists reduces fat stores most effectively [94, 113, 114]. Many epidemiological studies have shown the strong relationships between *β*3-adrenoceptor polymorphisms (mainly Trp54Arg), hypertension, metabolic syndrome, and obesity [78, 94, 113–117] (Table 4).

### 4.4. Confounding Variables Affecting the Relationships of *β*-Adrenoceptor Polymorphisms with Obesity, Hypertension, and Diabetes (Table 5)

Tables 2, 3, 4, and 5 show the discordant contributions of *β*-adrenoceptor polymorphisms to hypertension, metabolic syndrome (type 2 diabetes), and obesity. Table 5 summarizes factors which might explain the discrepancy of published data. Further, haplotypes of polymorphisms have strong influence on *β*-adrenoceptor function in each polymorphism [20, 58, 59, 105–107].

## 5. Conclusions

The role of the sympathetic nervous system *β*2- and *β*3-adrenoceptor polymorphisms in hypertension, metabolic syndrome (diabetes mellitus), and obesity is discussed through a literature review. Sympathetic nervous system activity and *β*-adrenoceptor polymorphisms (mainly *β*2- and *β*3-adrenoceptor polymorphisms) might contribute to the onset and maintenance of hypertension, metabolic syndrome, and obesity; however, the findings have been discordant. Further, few studies have been performed to evaluate the relationship between *β*2- and *β*3-adrenoceptor polymorphisms and sympathetic nervous system activity in the same study. A better understanding for the relationships of genetic background (polymorphisms) with sympathetic nervous system activity as the cause for hypertension (blood pressure elevation), metabolic syndrome (insulin resistance), and obesity (weight gain) might help for clinical treatment for obesity-related hypertension and metabolic syndrome. In fact, a number of studies have investigated genetic polymorphisms as determinants of cardiovascular response to antihypertensive drug therapy [103, 104]. But further research on gene-drug interactions is necessary. In addition, to clarify the pathogenesis and mechanisms may lead to the prevention of hypertension and metabolic syndrome in obesity.

## Figures and Tables

**Table 1:** Comparisons of adrenergic receptor subtypes.

Receptor type	Agonist potency order	Action sites	Functions
*α* *1-adrenoceptor *	norepinephrine≥	*blood vessels* of skim, gastrointestinal, kidney	*vasoconstriction*
	epinephrine⋙	ureter, uterus, urethral sphincter, bronchioles	smooth muscle contraction,
	isoprenaline	urinary bladder,	contraction,
		iris, blood vessels of erectile tissue,	smooth muscle relaxation,
		*heart muscle,*	*positive ionotropic effect*
		salivary gland,	increase in secretion,
		*adipose* *tissue, liver*	*glycogenolysis and gluconeogenesis,*
		sweat glands,	increase in secretion,
		*kidneys*	*Na reabsorption*

**α*2-adrenoceptor*	epinephrine>	pancreas and	*inhibition of insulin secretion, *
	norepinephrine⋙		*induction of glucagon release, and*
	isoprenaline	gastrointestinal tract	contraction of sphincters

**β*1-adrenoceptor*	isoprenaline>	*heart,*	*increase cardiac output, *
	Norepinephrine>	*kidneys* (juxtaglomerular cells),	*increase renin release, and *
	Epinephrine	*adipose tissue*	*lipolysis*

**β*2-adrenoceptor*	isoprenaline>	Bronchi,	smooth muscle relaxation,
	epinephrine≫	urinary sphincter, bladder wall,	smooth muscle relaxation,
	norepinephrine	*skeletal muscle, *	*dilate arteries *
		*adipose tissue, liver*	*glycogenolysis and gluconeogenesis,*
		gastrointestinal tract,	contract sphincters,
		salivary glands,	thickened secretions,
		mast cells, and	inhibit histamine release, and
		*kidneys (juxtaglomerular cells)*	*increase renin release*

**β*3-adrenoceptor*	isoprenaline> norepinephrine = epinephrine	*adipose tissue*	*enhancement of lipolysis*

**Table 2:** Arg16Gly, *β*2-adrenoceptor polymorphisms: association with hypertension, metabolic syndrome (type2 diabetes: (DM)), and obesity.

Authors	Year	Populations	Subjects	Associations with the polymorphism
Large et al. [40]	1997	Swedish	140 Caucasian women with a wide range of obesity	Obesity
The Quebec Family Study [41]	2000	Canada	Caucasian men and women	Obesity, hyperlipidemia
Hayakawa et al. [42]	2000	Japanese	210 Japanese men from a population	No association with obesity
Jia et al. [43]	2000	USA	Caucasians (298 hypertensive versus 298 normotensive subjects)	No association with hypertension
Xie et al. [44]	2000	USA	Black and white Americans (including normotensive and hypertensive subjects)	No associations with hypertension
Candy et al. [45]	2000	English	England Black African men (including 192 hypertensive and 123 normotensive men)	No association with hypertension
Cockcroft et al. [46]	2000	Caucasian	127 young normotensive men	Forearm vascular responses (hypertension)
Meirhaeghe et al. [47]	2000	French	1195 middle-aged Caucasian from the urban population	Obesity, if subjects carry Gln27Gln
				
Kato et al. [48]	2001	Japanese	842 hypertensive and 633 normotensive subjects	BP levels (hypertension) in normotensives
Bengtsson et al. [49]	2001	Swedish	Hypertensive patients with and without type 2 DM	Hypertension in subjects with DM
The Bogalusa Heart Study [50]	2002	USA	1151 Caucasian and Black Africans children (including boys and girls)	Weight gain in males
Kim et al. [51]	2002	Korean	type 2 DM patients	Obesity, DM, hyperlipidemia
Chang et al. [52]	2002	Taiwanese	type 2 DM patients	Type 2 DM
Van Rossum et al. [53]	2002	Dutch	286 subjects with a significant weight gain over 7 years including men and women	Weight gain in men, but not in women
The HERITAGE family study [54]	2003	Canada	Sedentary black and white women	Lower fat in obese white women
Pereira et al. [20]	2003	Brazilian	1576 ethnically mixed population (including men and women)	Systolic BP, BMI
The Olivetti heart study [55]	2004	Italian	993 middle-aged men regardless of BP levels or BMI	No association with obesity or hypertension
Ikarashi et al. [56]	2004	Japanese	type 2 diabetic patients	Association with IR
Tafel et al. [57]	2004	Germany	extremely obese children	No association with obesity
Ellsworth et al. [58]	2005	USA	Black and white American men and women	BMI (obesity) in only men
Trombetta et al. [59]	2005	Brazilian	Brazilian healthy women	Hypertension (blunted forearm vasodilation response)
Masuo et al. [21]	2005	Japanese	Nonobese, normotensive men	Weight gain, BP elevation, obesity-HT
Masuo et al. [60]	2005	Japanese	Nonobese, normotensive men	Insulin resistance
Masuo et al. [61, 62]	2006	Japanese	Normotensive men (including nonobese and obese men)	Weight gain, blunted leptin-sympathetic axis
Kurabayashi et al. [63]	2006	Japanese	PCOS patients	Association with high prevalence of PCOS Accompanying IR
Gjesing et al. [64]	2007	Dutch	7808 white subjects	No association with hypertension or obesity
Masuo et al. [65]	2007	Japanese	219 nonobese, normotensive men	Association with high SNA followed by IR

BP: blood pressure; BMI: body mass index; HT: hypertension; DM: diabetes mellitus; IR: insulin resistance; PCOS: polycystic ovary syndrome; SNA: sympathetic nervous activity.

**Table 3:** Gln27Glu, *β*2-adrenoceptor polymorphisms: association with hypertension, metabolic syndrome (type2 diabetes (DM)), and obesity.

Authors [reference number]	Year	Populations	Subjects	Associations with the polymorphism
Large et al. [40]	1997	Swedish	Caucasian women with a wide range of obesity	Association with obesity
Echwald et al. [66]	1998	Danish	Caucasian juvenile-onset obese men	No association with obesity
Hellström et al. [67]	1999	Swedish	Caucasian men and women	Association with obesity only in women
Kortner et al. [68]	1999	German	Caucasian with morbid obesity	No association with obesity
Xie et al. [44]	2000	USA	Black and white Americans	No associations with hypertension
The Quebec Family Study [41]	2000	Canada	Caucasian men and women	Association with obesity and hyperlipidemia
Hayakawa et al. [42]	2000	Japanese	210 Japanese men from a population	No association with obesity
Candy et al. [45]	2000	England	Black African men (including 192 hypertensive and 123 normotensive men)	No association with hypertension
Meirhaeghe et al. [47]	2000	French	1195 middle-aged Caucasian in the urban population	Association with obesity in men
Kato et al. [48]	2001	Japanese	842 hypertensive and 633 normotensive subjects	Association with BP levels (hypertension) in NT
Kawamura et al. [69]	2001	Japanese	Japanese-Americans	No association with obesity or DM
Ukkola et al. [70]	2002	USA	12 pairs of twins, Caucasians	Association with weight gain (obesity)
Kim et al. [51]	2002	Korean	Patients with type 2 DM	Association with obesity, DM, and hyperlipidemia
Gonzalez-Sanchez et al. [71]	2003	Spanish	666 Caucasian-based study (including men and women)	Association with obesity only in men
The HERITAGE family study [49]	2003	Canada	Sedentary black and white men	Association with lower fat in obese white men
Pereira et al. [20]	2003	Brazilian	1576 ethnically mixed population (including men and women)	No association with systolic BP or BMI
The Olivetti heart study [55]	2004	Italian	993 middle-aged men (regardless of BP levels or BMI)	No association with obesity or hypertension
Tafel et al. [57]	2004	Germany	Extremely obese children	No association with obesity
Masuo et al. [21]	2005	Japanese	Nonobese, normotensive men	Association with BP elevation, but no association with IR
Trombetta et al. [59]	2005	Brazilian	Brazilian healthy women	Association with hypertension (blunted forearm vasodilation response)
Kurabayashi et al. [63]	2006	Japanese	PCOS women	Association with high prevalence of PCOS accompanying IR
Gjesing et al. [64]	2007	Dutch	7808 white subjects	No association with hypertension or obesity
Masuo et al. [65]	2007	Japanese	219 nonobese, normotensive men	No association with IR

BP: blood pressure; BMI: body mass index; DM: diabetes mellitus; NIDDM: noninsulin-dependent diabetes mellitus; IR: insulin resistance; PCOS: polycystic ovary syndrome; NT: normotensive subjects.

**Table 4:** Trp64Arg, *β*3-adrenoceptor polymorphisms: association with hypertension, metabolic syndrome (type2 diabetes (DM)), and obesity.

Authors [reference number]	Year	Populations	Subjects	Associations with the polymorphism
Clement et al. [76]	1995	French	185 subjects with morbid obesity and	Increased capacity of weight gain
			94 subjects with normal weight	
Widen et al. [77]	1995	Finns	335 subjects including 207 non-DM and 128 patients with NIDDM	Insulin resistance
Walston et al. [78]	1995	Pima Indians	390 with NIDDM and 252 without NIDDM	Association with the early onset of DM2
Fujisawa et al. [79]	1996	Japanese	Patients with NIDDM	Type 2 DM, weight gain (obesity)
Silver et al. [80]	1996	Nauruans	65 obese subjects with NIDDM	No association with DM2 or IR
Fujisawa et al. [81]	1997	Japanese	Essential hypertension patients	No association with IR during hyperinsulinemia euglycemic glucose clamp
Sakane et al. [82]	1997	Japanese	131 obese women versus 218 controls	Association with IR and obesity
Rissanen et al. [83]	1997	Finns	110 with NIDDM, 183 with IR, and 82 controls	No association with NIDDM or IR
McFarlane-Anderson et al. [84]	1998	Jamaican	Population study	Association with hyperglycemia only in women, but not in men
Gracía-Rubi et al. [85]	1998	American	Postmenopausal women	Association with IR
Janssen et al. [86]	1998	Dutch	Postmenopausal women	Association with IR
Shiwaku et al. [87]	1998	Japanese	Moderate overweight men	No association with obesity
Ongphiphadhanakul et al. [88]	1999	Thais	76 men and 135 women	No association with IR assessed by fasting insulin/glucose ratio
Pulkkinen et al. [89]	1999	Finns	185 untreated non-DM and 119 untreated NIDDM	No association with IR or CHD in both non-DM and NIDDM
Christiansen et al. [90]	1999	Danish	196 dizygotic twins	Association with lower insulin secreting capacity
Kawamura et al. [69]	1999	Japanese-American	Japanese living in USA versus living in Japan	Similar distribution between Japanese-America and Japanese-Japanese. Association with IR in subjects with impaired oral glucose tolerance test.
Stangl et al. [91]	2001	German	1000 with CHD and 1000 controls	No association with prevalence of CHD or IR
Strazzullo et al. [92] (The Olivetti Prospective Heart Study)	2001	Italian	979 population study	No association with IR observed in HOMA-IR
Ishii et al. [93]	2001	Japanese	196 young normoglycemic men, 186 old normoglycemic men, and 122 old hyperglycaemic men	No association with IR or NIDDM
Kurokawa et al. [94]	2001	Japanese	meta-analysis in 6582 subjects	BMI (obesity)
Ochoa et al. [95]	2004	Spanish	185 obese and 185 nonobese children	BMI (obesity)
Porto et al. [96]	2004	Argentina	121 NT and 54 HT from 934 high school students	Association with central obesity, but no association with IR
Tsai et al. [97]	2004	Taiwanese	299 pregnant women	No association with gestational IR
Ellsworth et al. [58]	2005	USA	1179 African-Americans and white-Americans	BMI (obesity)
Masuo K, et al. [21]	2005	Japanese	Nonobese, normotensive men	BP elevation
Masuo et al. [62]	2006	Japanese	55 obese normotensive men	Weight gain (obesity), BP elevation (hypertension)
Højlund et al. [98]	2006	Danish	10 male twins	No association between heterozygous for Trp64Arg and IR or NIDDM
Tamaki et al. [99]	2006	Japanese	1416 population study without HT, DM, or hyperlipidemia	No association with metabolic syndrome
Morcillo et al. [100]	2008	Spanish	1020 population study	Join association of alleles of -75A and Arg64 with the risk of DM
Gjesing et al. [101]	2008	Danish	7605 population study	Association with NIDDM and IR, but no association with obesity
Dunajska et al. [102]	2008	Polish	284 postmenopausal women	No association with metabolic syndrome

BP: blood pressure; BMI: body mass index; DM: diabetes mellitus; NIDDM: noninsulin-dependent diabetes mellitus; DM2: type 2 diabetes mellitus; IR: insulin resistance.

**Table 5:** Confounding variables considered to cause the discrepancy of the relationships between *β*-adrenoceptor polymorphisms and phenotypes of hypertension and metabolic syndrome in obesity.

Variables [reference number]	Findings in the studies
Severity of obesity [16, 57, 62, 76, 95]	In lean subjects, *β*2-AR polymorphisms linked to obesity and obesity-related hypertension, but in obese subjects, *β*2- and *β*3-AR polymorphisms relate to obesity and obesity-related hypertension.
	Morbid obesity is linked with *β*3-AR polymorphisms, but overweight or mild obesity is not associated with those.

Gender differences [71]	Interaction between *β*1- and *β*2-AR polymorphisms with changes in BMI was observed in men only, while in women an interaction between *β*1- and *β*3-AR polymorphisms was observed in a longitudinal over a 24-year period large cohort study.

Ethnic difference [103, 104]	Distributions of *β*-AR polymorphisms are different in 8 different ethnic populations.

Haplotype [20, 58, 59, 105–107]	Functions expressed of *β*-AR polymorphisms are different due to the other *β*-AR polymorphisms.

AR: adrenoceptor; BMI: body mass index.
